# The Circadian Clock Gene, *TaPRR1*, Is Associated With Yield-Related Traits in Wheat (*Triticum aestivum* L.)

**DOI:** 10.3389/fpls.2020.00285

**Published:** 2020-03-12

**Authors:** Han Sun, Wenping Zhang, Yongzhen Wu, Lifeng Gao, Fa Cui, Chunhua Zhao, Zhiai Guo, Jizeng Jia

**Affiliations:** ^1^College of Agriculture, Ludong University, Yantai, China; ^2^Key Laboratory of Molecular Module-Based Breeding of High Yield and Abiotic Resistant Plants in Universities of Shandong, Ludong University, Yantai, China; ^3^National Key Facility for Crop Gene Resources and Genetic Improvement, Institute of Crop Science, Chinese Academy of Agricultural Sciences, Beijing, China; ^4^Key Laboratory of Plant Molecular Physiology, Institute of Botany, Chinese Academy of Sciences, Beijing, China

**Keywords:** *TaPRR1*, haplotype, circadian rhythm, association analysis, yield-related traits, genetic differentiation

## Abstract

Timing of flowering is crucial for the transformation from vegetative to reproductive growth in the important food crop, wheat (*Triticum aestivum* L.). The circadian clock is a central part of photoperiod regulation, with Pseudo-Response Regulators (PRRs) representing key components of circadian networks. However, little is known about the effects of PRR family members on yield-related traits in crop plants. In this study, we identified polymorphisms and haplotypes of *TaPRR1*, demonstrating that natural variations in *TaPRR1* are associated with significant differences in yield-related traits including heading date, plant height and thousand grain weight. *TaPRR1-6A-Hapla* showed an earlier heading date, advanced by 0.9 to 1.7%. *TaPRR1-6B-Hapla* and *TaPRR1-6D-Hapla* displayed reduced plant height and increased thousand grain weight of up to 13.3 to 26.4% and 6.3 to 17.3%, respectively. Subcellular localization and transcriptional activity analysis showed that TaPRR1 is a nuclear localization protein with transcriptional activity controlled by an IR domain. The expression profiles of *TaPRR1* genes over a 48-h period were characterized by circadian rhythms, which had two peaks under both short- and long- day conditions. In addition, geographical distribution analysis indicated higher distribution frequencies of *TaPRR1-6A-Hapla*, *TaPRR1-6B-Haplb*, and *TaPRR1-6D-Haplb* in different agro-ecological production zones. Furthermore, analysis of molecular variance of the distribution frequency of *TaPRR1* haplotypes suggested significant differences in haplotype distribution frequency between landraces and modern cultivars. Our study provides a basis for in-depth understanding of *TaPRR1* function on yield-related traits in wheat, as well as establishing theoretical guidance for wheat molecular marker-assisted breeding.

## Introduction

Wheat (*Triticum aestivum* L., AABBDD) is one of the most important food crops and sources of calories for humans. During growth, the timing of flowering (heading date in cereal crops) is key for the transformation of wheat from vegetative to reproductive growth. Photoperiod response genes not only affect the growth phase, but also play an important role in the adaptability and crop yield of wheat. In the photoperiod pathway, the photoreceptor senses a light signal and transmits it to the circadian clock, which generates biological rhythms and exports them to downstream-regulated genes. The circadian clock is a central part of photoperiod regulation, the spontaneous mechanisms of biological rhythms with a cycle of nearly 24 h (Niinuma et al., 2007). It is an internal time-keeping mechanism that provides an adaptive advantage by enabling organisms to anticipate daily changes and orchestrate biological processes accordingly. Circadian regulated Pseudo-Response Regulators (PRRs) are key components of circadian networks in plants (Farré and Liu, 2013). PRRs were first described in *Arabidopsis* and have been demonstrated to play a key role in the regulation of the circadian oscillator and clock output processes (Makino et al., 2000; Strayer et al., 2000; Harmer, 2009).

*PRR1* (*Pseudo-response regulator 1*), also known as *TOC1* (*TIMING OF CAB EXPRESSION 1*), is a member of the *PRR* gene family. The *Arabidopsis PRR* gene family consists of five members (*PRR9*, *PRR7*, *PRR5*, *PRR3*, and *PRR1*), all of which are regulated by the circadian clock, but each of which peaks at a different time of day (Matsushika et al., 2000). *PRR1* together with two MYB transcription factors, *CCA1* (*CIRCADIAN CLOCK-ASSOCIATED 1*) and *LHY* (*LATE ELONGATED HYPOCOTYL*), constitute the main feedback loop of the central oscillator. Reciprocal transcriptional regulation between *PRR1*, *CCA1*, and *LHY* was proposed to be essential for circadian rhythmicity, with *PRR1* repressing the transcription of *CCA1* and *LHY* (Harmer et al., 2000; Alabadí et al., 2001; Gendron et al., 2012; Huang et al., 2012). *Arabidopsis prr1-1* exhibits a shortened period phenotype in multiple rhythms and altered circadian clock regulation of multiple outputs during development (Somers et al., 1998; Strayer et al., 2000). Silencing or overexpression of *PRR1* abolishes photoperiod rhythmicity and changes the light sensitivity in the control of hypocotyl elongation, indicating that *PRR1* has dual functionality, not only in the integration of light signals to control circadian rhythm, but also in the photomorphogenic response (Más et al., 2003).

*PRR* gene family members also play an important role in wheat. *Ppd-1* (*TaPRR37*), the famous gene conferring photoperiod insensitivity in “Green Revolution” cultivars, belongs to the *PRR* gene family and has made a significant contribution to wheat yield improvement (Laurie, 1997; Worland and Snape, 2001; Turner et al., 2005; Beales et al., 2007). Genetic and epigenetic variations in the *Ppd-1* gene have been used to develop specific molecular markers for polymorphic genetic loci and classify *Ppd-1* haplotypes. Genetic variations in the *Ppd-1* gene not only affect the photoperiod sensitivity of wheat, but are also associated with important agronomic traits, including plant height and thousand grain weight (Beales et al., 2007; Wilhelm et al., 2009; Guo et al., 2010; Seki et al., 2011; Díaz et al., 2012; Nishida et al., 2013; Sun et al., 2014; Arjona et al., 2018). *TaPRR73* is a paralog of *Ppd-1* known to affect heading date and plant height in wheat. Overexpression of *TaPRR73* accelerates flowering under long photoperiods (Zhang et al., 2016). *TaPRR1* has also been reported in wheat. MicroRNA tae-miR408 functions in wheat heading time by mediating the expression of *TaPRR1*, one of its targets. Transgenic plants with knockdown of *TaPRR1* and overexpression of tae-miR408 show an early heading phenotype (Zhao et al., 2016). Although some progress has been made in functional studies of wheat *PRR* family members, studies defining the influence of the circadian clock core member *TaPRR1* on yield-related traits, as well as functional polymorphic sites, characteristics of haplotype classification and geographical distribution, and the development and application of molecular markers, are still relatively lacking.

In our current study, we further analyzed the functions of the circadian clock gene *TaPRR1*. We discovered and evaluated haplotypes of the wheat *TaPRR1* gene. Association analysis using natural populations in multiple environments indicated that *TaPRR1* is significantly associated with important agronomic traits, including plant height, thousand grain weight, and heading time. Geographical distribution analysis indicated that *TaPRR1* haplotypes show distinctive characteristics in different agro-ecological production zones. Furthermore, assessment of the distribution frequency of the different haplotypes suggested that *TaPRR1* genes have undergone selection during the wheat breeding process. Our study provides not only a theoretical basis for understanding the functional mechanisms of the wheat *PRR* gene family, but also some theoretical guidance for wheat molecular marker-assisted breeding.

## Materials and Methods

### Plant Material

Hexaploid wheat (*T. aestivum*) cultivar “Chinese Spring” (CS) and diploid varieties *T. urartu* UR102 (AA), *Aegilops Tauschii* Y215 (DD), and *A. Speltoides* Y2022 (SS) were used for *TaPRR1* gene cloning. Twenty-eight accessions with wide variations in heading date and yield-related traits (Supplementary Table S1) were used to detect naturally occurring variations in *TaPRR1* gene sequences. A total of 177 wheat accessions, including 129 accessions from the Chinese wheat Mini Core Collection (MCC) (Hao et al., 2011), 40 germplasm resources preserved in the Chinese Academy of Agricultural Sciences (CAAS, Beijing, China), and eight synthetic hexaploid wheat lines, were screened for *TaPRR1* genotype and used for association analysis of *TaPRR1* (Supplementary Table S2). The above materials were separately planted in Luoyang (111.6°E, 33.8°N), Xinxiang (113.8°E, 35.2°N), Changping (116.2°E, 40.2°N), Beijing (116.2°E, 39.5°N), Bashang (115.7°E, 41.7°N), Jiaozuo (113.2°E, 35.1°N), and Shunyi (116.5°E, 40.1°N) during the years 2006–2015. The characteristics of these locations are described in Supplementary Table S3. Six plants showing even growth were selected for each genotype for investigating agronomic traits including effective tiller number (ETN), plant height (PH), spike length (SL), total number of spikelets per spike (TNSS), number of grains per spike (NGS), thousand grain weight (TGW), and heading date (HD). For geographic distribution analysis, a total of 177 wheat accessions spanning a wide geographical area were evaluated (Supplementary Table S2). The Chinese accessions comprised landraces and modern wheat cultivars derived from the 10 major agroecological wheat regions (Jin, 1983) and were selected from the Chinese wheat MCC (Hao et al., 2011). Accessions chosen from Asia, Europe, North America, South America, and Oceania were representative accessions with a rich diversity of photoperiod responses selected from both the MCC and CAAS. For haplotype frequencies analysis, a total of 124 wheat accessions, including 60 modern cultivars, 56 landraces, and eight synthetic hexaploid wheat lines, were investigated (Supplementary Table S4).

### Subcellular Localization

Coding sequences of *TaPRR1-D1* were cloned into *p2GWF7* using Gateway cloning technology (Invitrogen, United States) to generate the TaPRR1-D1-GFP construct (Karimi et al., 2002). Similarly, the coding sequence of *OsMADS15* was cloned into *p2GWR7* to generate OsMADS15-RFP as a nuclear marker. TaPRR1-D1-GFP and OsMADS15-RFP constructs were co-transformed into rice protoplasts as described previously (Bart et al., 2006). After 20–24 h of incubation at 28°C, green fluorescent protein (GFP) and red fluorescent protein (RFP) fluorescence were observed using a confocal laser-scanning microscope (Carl Zeiss LAM510, Germany).

### Transactivation Activity Assay

TaPRR1-D1 proteins with no functional domain (1–29 aa), CheY-like REC (receiver) domain (1–176 aa), IR domain (186–431 aa), CCT domain (426–520 aa), IR-CCT domain (186–520 aa), and full length TaPRR1-D1 (1–520 aa) were subcloned into *pGBKT7* (Clontech) to generate DNA binding domain fusion constructs. For transactivation activity assay, vectors carrying the target segment were transformed into yeast strain AH109 (Clontech) according to the manufacturer’s protocols. Yeast cells transformed with the indicated vectors were diluted and then dropped onto tryptophan-negative (–Trp) and tryptophan-, histidine- negative (–Trp/–His) synthetic dropout (SD) media, respectively. Plates were incubated at 30°C until colonies appeared.

### Molecular Marker Development for *TaPRR1*

Three cleaved amplified polymorphic sequence (CAPS) markers, P78, P272, and P321, for *TaPRR1-A1* were designed to identify polymorphisms in the 5′UTR, intron 1 and exon 2, respectively. Marker P78 was used to detect a single nucleotide polymorphism (SNP) locus in the 5′UTR. This assay used nested PCR with two pairs of primers. Products generated by PCR using the first primer pair, P78-1F and P78-1R, were used as templates for amplification with the second pair, P78-2F and P78-2R. PCR products were then digested by *Bsp*1286I (GDGCH/C) at 37°C for 3 h. If the product could not be digested by *Bsp*1286I, base A was located at −78 bp upstream of the initiation codon ATG. If the product could be digested into two fragments (114 bp and 294 bp), base G was located at this SNP locus. Using the same method, markers P272 and P321 were designed to detect polymorphisms in intron 1 and exon 2, respectively. Two allele-specific PCR (AS-PCR) markers, P1055 and P1230, were developed to detect variations in *TaPRR1-B1*. The CAPs marker P1941 was designed to identify polymorphism in the *TaPRR1-D1* promoter region. This assay also used nested PCR with two pairs of primers, P1941-1F/1R and 2F/2R. The PCR product was digested by *Bts*CI (GGATGNN/) at 50°C for 3 h. If the product could not be digested by *Bts*CI, base G was located at −1941 bp upstream of ATG. If the product could be digested into two fragments (116 bp and 315 bp), base A was located at this SNP locus (Supplementary Table S5). All PCR primers are listed in Supplementary Table S6.

### Expression Analysis

The expression experiment was conducted under short-day (9 h: 15 h, light: dark) and long-day (15 h: 9 h, light: dark) conditions with samples collected every 3 h during a 48-h period from 14-day-old seedlings of wheat cultivars “Chinese Spring” (CS, *Hapl*a/*Hapl*b*/Hapl*b), “Yanzhan1” (YZ1, *Hapl*a/*Hapl*a*/Hapl*a), and “Hussar” (*Hapl*a/*Hapl*b*/Hapl*a). Four plants were pooled at each time-point and three biologically independent replications were performed for each genotype. Total RNA was extracted using RNAiso Plus (Takara, Ohtsu, Shiga, Japan). DNA was removed by digestion with DNase I (Fermentas, Ontario, Canada), and first-strand cDNA was synthesized using Moloney Murine Leukemia Virus (M-MLV) reverse transcriptase (Invitrogen). Quantitative reverse transcription PCR (RT-qPCR) was conducted using SYBR^®^ *Premix Ex Taq^TM^* (Takara) on an ABI PRISM 7300 (Applied Biosystems, Foster City, CA, United States). Expression levels of *TaPRR1* genes were standardized against that of the housekeeping gene *glyceraldehyde-3-phosphate dehydrogenase* (*GAPDH*), and quantitative data were analyzed using the 2^–ΔΔCt^ method (Livak and Schmittgen, 2001).

### Phylogenetic Analysis

Amino acid sequences of the *PRR* gene family were obtained from the Protein database^1^. Multiple sequence alignment was performed using ClustalW. A neighbor-joining tree was reconstructed from alignment of 15 wheat PRR protein sequences using MEGA 6.06 with 1,000 bootstrap replicates and the Jones-Taylor-Thornton (JTT) model (Tamura et al., 2013).

### PCR Primers, Sequence Analysis and Statistical Analysis

Primers were designed using Primer Premier 5.0 software (Supplementary Table S6). Sequence alignment and SNP detection were carried out using DNASTAR Lasergene 7.1.0 (DNASTAR). *Cis*-regulatory elements were predicted using PLACE (Higo et al., 1999). Significance tests were performed using SPSS Statistics 18.0. Tukey’s test was used to determine statistical differences by one-way ANOVA. Association analysis was performed by retrieving genotype data spanning 1-Mb regions upstream and downstream of *TaPRR1* using the “Axiom Wheat 660K Genotyping Array” scanning 681 natural population (Supplementary Table S7)^2^. The phenotype data was collected in Changping (116.2°E, 40.2°N) during the years 2012–2013. Sequence variants with minor allele frequency (MAF) ≥0.05 were retained, and their associations with yield-related traits were tested using a general linear model that accounts for familial relatedness in Tassel 5.0 (Bradbury et al., 2007). Pairwise linkage disequilibrium analysis was performed using LDheatmap packages (Shin et al., 2006). Analysis of molecular variance (AMOVA) was performed using ARLEQUIN version 3.5^3^ with an *F*-statistic to assign the genetic variation to either divergence among or variation within groups. Bisulfite sequencing data of DNA methylation levels of *TaPRR1* in two-week-old CS leaf tissue at the three-leaf stage was available under project ID SRP133674 (Appels et al., 2018). Histone modification levels of *TaPRR1* in 10-day-old *in vitro* seedlings were available under SRA study PRJNA420988 (SRP126222) (Appels et al., 2018). The integrated and visualized data was downloaded from The Triticeae Multi-omics Center website^4^.

## Results

### Isolation of *TaPRR1* Homeologs and Phylogenetic Analysis of the *PRR* Gene Family in Wheat

Primer pair TaPRR1-F1/R1 specific for full-length *TaPRR1* was designed and used to amplify three homeologs of *TaPRR1* genes (*TaPRR1-A1*: TraesCS6A02G227900; *TaPRR1-B1*: TraesCS6B02G253900; and *TaPRR1-D1*: TraesCS6D02G207100) from hexaploid wheat cultivar “Chinese Spring” (*T. aestivum*, AABBDD). The full-length *TaPRR1* genes of diploid A, B, and D genome donors UR102 (*T. urartu*, AA), Y2022 (*A. speltoides*, SS), and Y215 (*A. tauschii*, DD) were also obtained. Comparison of gene sequences from hexaploid and diploid accessions showed that different sequences from CS correspond to A, B, and D genomes, respectively (Supplementary Table S8).

**FIGURE 1:**
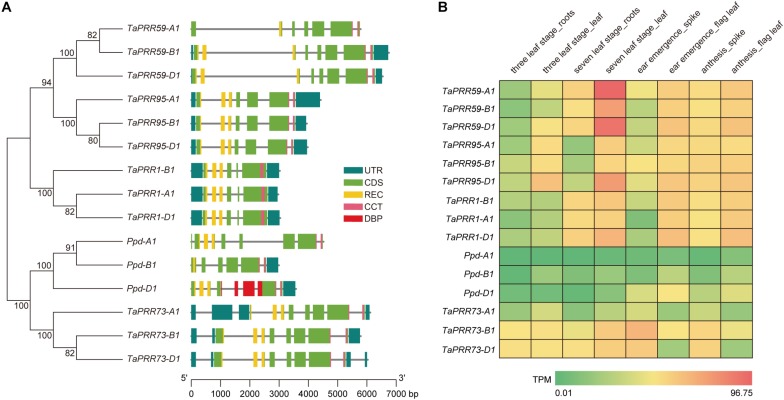
Phylogenetic relationships and expression profiles of the *PRR* gene family in wheat. **(A)** Phylogenetic analysis of the *PRR* gene family in wheat. A neighbor-joining tree was reconstructed from alignment of 15 wheat PRR protein sequences using MEGA 6.06 and the Jones-Taylor-Thornton model. Numbers above the branches represent bootstrap support based on 1,000 bootstrap replicates. Gene structures of *TaPRR* genes. UTR and CDS are represented by blue and green rectangles, respectively. Conserved domains of the proteins are also marked on the gene structure. REC, CCT, and DBP domains are represented by yellow, purples, and red respectively. Genes and their constituent parts can be inferred using the scalebar at the bottom. **(B)** Heat map of *in silico* expression profiles of *PRR* gene family members in different organs at different growth stages of “Chinese Spring”. The expression data were generated from the expVIP database (http://www.wheat-expression.com/). The color scale represents TPM (Transcripts Per Kilobase of exon model per Million mapped reads) value. Green indicates low levels of transcript abundance, while red indicates high levels.

*TaPRR1* is a member of the *PRR* gene family. To explore the genetic relationships among *PRR* gene family members, we reconstructed a neighbor-joining tree (Figure 1A). The results showed that wheat PRR proteins could be divided into two clusters, which were further divided into five groups, each containing three homeologs from the wheat A, B, and D subgenomes. TaPRR1 showed the closest relationship with TaPRR95 and TaPRR59. Ppd-1 (TaPRR37) and TaPRR73 clustered in another branch of the tree. It is noteworthy that TaPRR1 and other *PRR* gene family members all contain two conserved domains, a REC domain and a CCT domain. The REC domain is a signal receiver domain and usually found N-terminal to a DNA binding effector domain (Marchler-Bauer et al., 2017). The CCT (CONSTANS, CO-like, and TOC1) domain is found in a number of plant proteins. It is about 45 amino acids long and contains a putative nuclear localization signal within the second half of the CCT motif (Marchler-Bauer et al., 2017). Remarkably the DBP (Duffy-antigen binding protein) domain is unique to the *Ppd-D1* gene.

Using expression data from the expVIP database^5^, we drew a heat map of *PRR* expression profiles at different developmental stages in different tissues (Figure 1B). The spatiotemporal expression patterns of *TaPRR1* were similar to those of *TaPRR59* and *TaPRR95*, but different from those of *Ppd-1* and *TaPRR73*. The expression level of *TaPRR1* in leaves (TPM value 25.32, 31.67, 43.47 for *TaPRR1-A1*, *-B1*, *-D1* at 7-leaf stage) was higher than that of roots (TPM value 18.75, 21.46, 25.30 for *TaPRR1-A1*, *-B1*, *-D1* at 7-leaf stage). During the reproductive growth period, the expression level of *TaPRR1* in flag leaves (TPM value 28.21, 28.90, 37.14 for *TaPRR1-A1*, *-B1*, *-D1* at ear emergence stage) was higher than that of spikes (TPM value 2.26, 7.20, 5.13 for *TaPRR1-A1*, *-B1*, *-D1* at ear emergence stage). The spatiotemporal expression patterns of *TaPRR1-A1*, *-B1*, and *-D1* were consistent. The expression level of *TaPRR1-D1* was higher than those of *TaPRR1-A1* and *-B1* (Figure 1B).

### Subcellular Localization and Transcriptional Activity Analysis of *TaPRR1*

**FIGURE 2:**
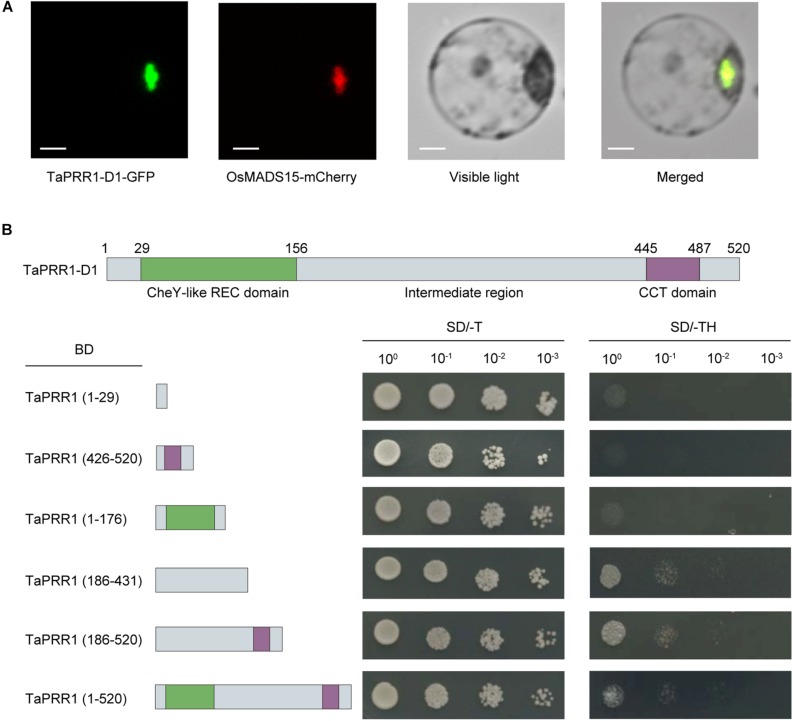
Subcellular localization and transcriptional activity analysis of TaPRR1. **(A)** Subcellular localization of TaPRR1. A fusion protein (TaPRR1-D1-GFP) and control (OsMADS15-RFP) were introduced into rice protoplasts. GFP and RFP fluorescence were observed using a confocal laser-scanning microscope. A nuclear marker protein, OsMADS15, fused with mCherry, was used as a positive control. Scale bars = 5 μm. **(B)** Transactivation activity assay. TaPRR1-D1 proteins with no functional domain (1–29 aa), CheY-like REC domain (1–176 aa), IR domain (186–431 aa), CCT domain (426–520 aa), IR-CCT domain (186–520 aa), and full length TaPRR1-D1 (1–520 aa) were assembled into *pGBKT7* vectors. Yeast cells transformed with the indicated vectors were diluted and then dropped onto tryptophan- negative (–Trp) and tryptophan-, histidine- negative (–Trp/–His) synthetic dropout (SD) media, respectively.

TaPRR1 contains a CheY-like REC domain at its N-terminus and a CCT motif at the C-terminus. The CCT motif contains a putative nuclear localization signal. Based on coding regions of the D subgenome member, we determined the subcellular localization of TaPRR1 by transient expression (Figure 2A). A nuclear marker protein, OsMADS15, fused with mCherry, was used as a positive control. The subcellular localization results showed overlapping GFP and RFP fluorescence signals in the nuclei, indicating that TaPRR1 is localized in the nucleus.

To further investigate whether TaPRR1 has transcriptional activation activity, we carried out a transcriptional activation assay based on coding regions of *TaPRR1-D1*. The results showed that the constructs fusing the full length or IR (intermediate region) domain of TaPRR1-D1 with BD (GAL4 binding domain) resulted in expression of reporter genes (Figure 2B). We observed no activation signal from fusion proteins containing only the CheY-like REC or CCT domain. These results suggested that TaPRR1 has transcriptional activity in its IR motif, which is similar to *Arabidopsis* PRR1 (Gendron et al., 2012). Taken together, these results demonstrated that TaPRR1 is a nuclear-localized protein with transcriptional activity controlled by the IR domain.

### Haplotype Analysis of *TaPRR1* and Molecular Marker Development

**FIGURE 3:**
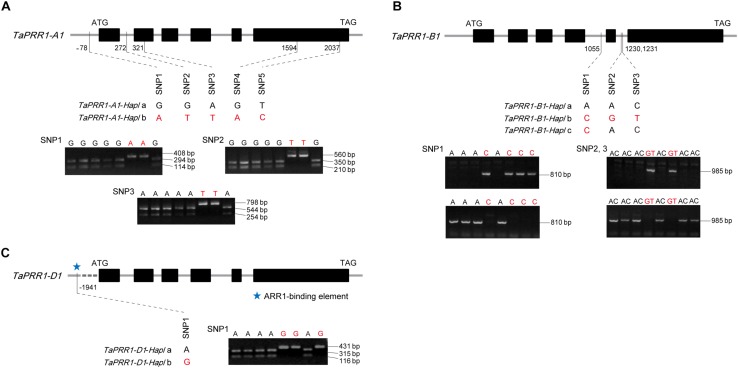
Gene structure, haplotypes, and molecular markers of *TaPRR1*. Taller rectangles represent the coding regions; shorter rectangles represent the candidate promoter, 5′ untranslated region (UTR), introns, and 3′UTR. **(A)** Haplotypes and molecular markers of *TaPRR1-A1*. Two haplotypes were identified for *TaPRR1-A1*. Three CAPS markers were developed based on SNP1-SNP3 with restriction endonucleases *Bsp*1286 I (SNP1 and SNP2) and *Btg*I (SNP3). **(B)** Haplotypes and molecular markers of *TaPRR1-B1*. Three haplotypes were identified for *TaPRR1-B1*, characterized by three SNPs located within intron 4 and intron 5. Two allele-specific PCR markers were developed to detect the variations of *TaPRR1-B1*. **(C)** Haplotypes and molecular markers of *TaPRR1-D1*. Two haplotypes were identified for *TaPRR1-D1*. A CAPS marker was developed based on SNP1 with restriction endonuclease *Bts*CI. The sizes of PCR products are shown on the right.

To analyze the polymorphism of *TaPRR1* and explore the relationship between natural variations of *TaPRR1* and agronomic traits, we analyzed the entire genomic fragment of *TaPRR1* in 28 cultivars with wide variations in HD and yield-related traits (Supplementary Table S1). Two haplotypes of *TaPRR1-A1* were characterized by five variations, all of which were SNPs and located upstream of the coding region, intron 1, exon 2, and exon 6, respectively (Figure 3A). Notably, SNP3-SNP5 were located in the exon region, but only SNP4 caused amino acid changes (from E to K). Three cleaved amplified polymorphic sequence (CAPS) marker were developed based on SNP1-SNP3 to identify the two haplotypes simultaneously (Figure 3A). For *TaPRR1-B1*, three haplotypes were identified, characterized by three SNPs located within intron 4 and intron 5, respectively. Two allele-specific PCR (AS-PCR) markers, P1055 and P1230, were developed to detect the variations (Figure 3B). For *TaPRR1-D1*, there was no nucleotide variation in the coding region. We only found a SNP located 1,941 bp upstream of the coding region, which resulted in two haplotypes of *TaPRR1-D1*. The CAPs marker P1941 was developed based on this polymorphism for diagnosing the *TaPRR1-D1* alleles of more accessions (Figure 3C). It is noteworthy that an ARR1-binding element (GGATT)^6^ was found at this SNP site and a single-base substitution mutation caused variations of this site, which might block its function.

### *TaPRR1* Exhibited a Diurnal Rhythm Expression Pattern

**FIGURE 4:**
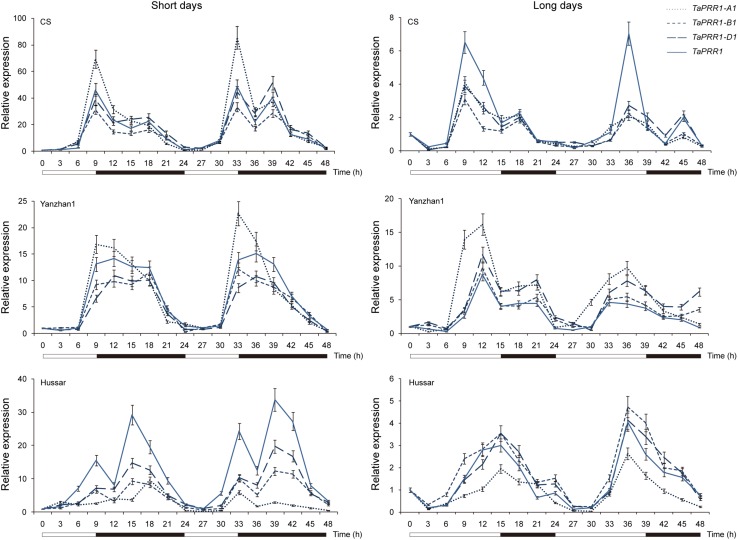
Relative expression levels of *TaPRR1* under short days (9 h light/15 h dark) and long days (15 h light/9 h dark) by quantitative RT-PCR. RNAs were sampled at 3-h intervals over a 48-h period. Means ± SD from three replicated experiments are shown. Open bars, daytime; closed bars, nighttime.

We analyzed the expression patterns of *TaPRR1* genes over a 48-h period in common wheat cultivars grown under short- and long- day conditions (Figure 4). Three representative cultivars, “Chinese Spring” (CS, *Hapl*a/*Hapl*b*/Hapl*b), “Yanzhan1” (YZ1, *Hapl*a/*Hapl*a*/Hapl*a), and “Hussar” (*Hapl*a/*Hapl*b*/Hapl*a) were selected for expression tests. Overall, the expression patterns of *TaPRR1* were characterized by diurnal rhythms, which had peaks at around 9 h after dawn and 18 h after dawn under both short- and long-day conditions. Specifically, “Hussar” showed different expression patterns compared to CS and YZ1. The first peak was higher than the second peak in CS and YZ1, whereas the second peak was higher in *TaPRR1-B1* and *TaPRR1-D1* of “Hussar” under short-day conditions. The peak appeared a few hours later in “Hussar” compared to CS and YZ1 under long-day conditions. The genotypes of *TaPRR1-B1* and *-D1* in “Hussar” were different from those in CS and YZ1, which may be the cause of the expression differentiation. In future studies, it will be of great significance to verify *cis*-regulatory elements/*trans*-transcriptional regulators to elucidate the detailed molecular mechanisms regulating *TaPRR1* expression.

### Association of *TaPRR1* Haplotypes With Agronomic Traits

**FIGURE 5:**
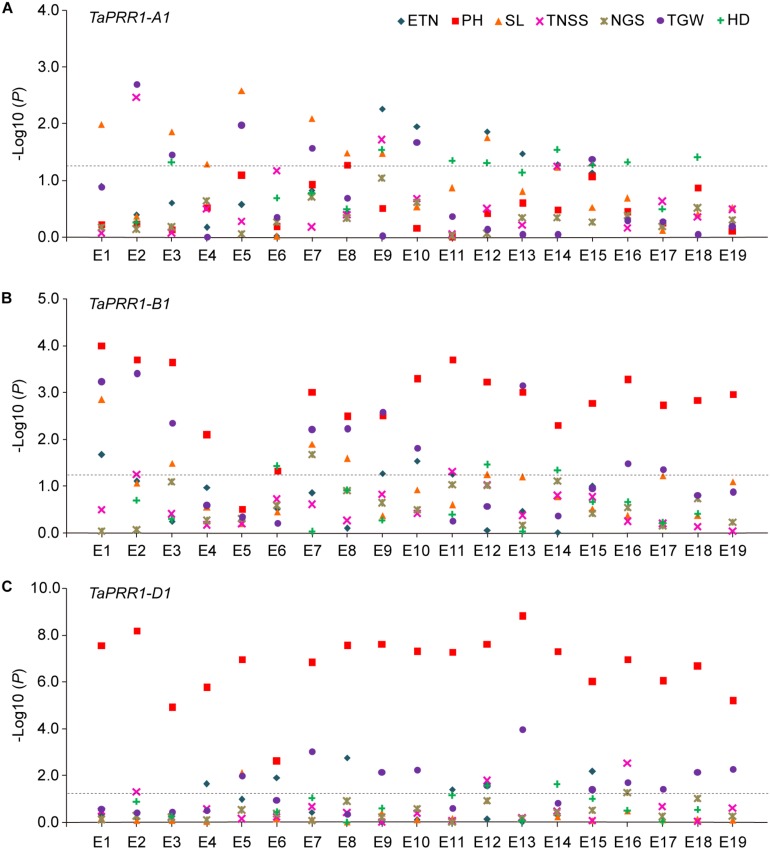
Association analysis of *TaPRR1* homeologs. **(A)** Association analysis of *TaPRR1-A1*. **(B)** Association analysis of *TaPRR1-B1*. **(C)** Association analysis of *TaPRR1-D1*. ETN, effective tiller number; PH, plant height; SL, spike length; TNSS, total number of spikelets per spike; NGS, number of grains per spike; TGW, thousand grain weight; HD, heading date. E1 to E19 indicate environments. Negative log_10_-transformed *P* values are plotted. Significance tests were performed using SPSS Statistics 18.0. Tukey’s test was used to determine statistical differences by one-way ANOVA. Black horizontal dotted line indicates the threshold value for significant associations (*P* < 0.05).

In order to investigate the association of *TaPRR1* haplotypes with yield-related traits, we performed an association analysis of each haplotype with seven yield traits using 177 accessions spanning 10 major agroecological wheat regions (Supplementary Table S2). Association analysis showed the homeolog-specific functions of *TaPRR1* (Figure 5). For *TaPRR1-A1*, there were weak associations between *TaPRR1-A1* and HD (seven environments), as well as SL (seven environments) (Figure 5A). However, *TaPRR1-B1* was significantly associated with PH in all environments except E5 (18 environments) and weakly associated with TGW (10 environments) (Figure 5B). Similarly, *TaPRR1-D1* was strongly associated with PH in all environments (19 environments) and weakly associated with TGW (10 environments) (Figure 5C).

We compared the phenotype variations of different *TaPRR1* haplotypes for the above agronomic traits. In general, for *TaPRR1*-*A1*, varieties with *Hapl*a showed earlier HD. It is noteworthy that *Hapl*b showed a longer SL compared to *Hapl*a (Supplementary Figure S1A). For *TaPRR1-B1* and *-D1*, accessions with *Hapl*a showed more favorable phenotypic traits, including shorter PH and higher TGW (Supplementary Figures S1B,C). Specifically, for *TaPRR1-A1*, the HD of *Hapl*a was about 1.83 to 3.45 days ahead, advanced by 0.9 to 1.7%. The SL of *Hapl*b increased by about 0.79 to 1.79 cm, representing an increase of 8.2 to 16.9% compared to *Hapl*a. For *TaPRR1-B1* and *-D1*, *Hapl*a reduced PH by about 12.37 to 23.60 and 12.89 to 30.26 cm, respectively, representing a decrease of 13.3 to 19.7% and 14.1 to 26.4% compared to *Hapl*b. *Hapl*a also increased TGW by about 3.17 to 6.85 and 2.28 to 5.78 g, representing an increase of 9.1 to 17.3% and 6.3 to 14.8%, respectively, compared to *Hapl*b.

**FIGURE 6:**
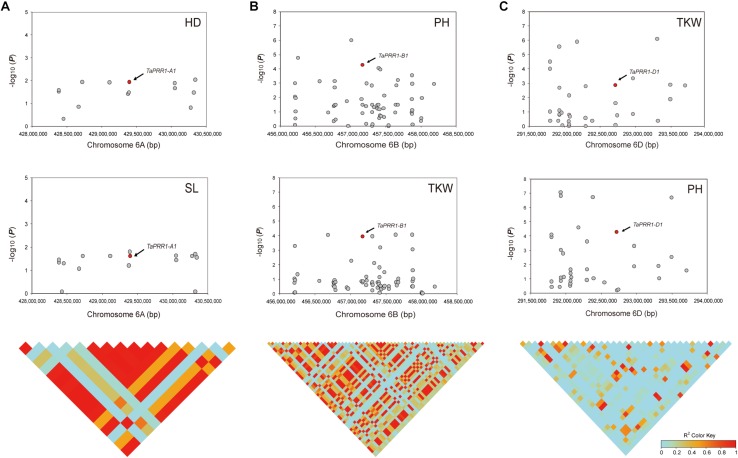
Association analysis of the 2-Mb region for *TaPRR1* in a panel of 681 natural population. **(A)** Association studies were performed for *TaPRR1-A1* with HD and SL. **(B)** Association studies were performed for *TaPRR1-B1* with PH and TGW. **(C)** Association studies were performed for *TaPRR1-D1* with PH and TGW. The lower diagram shows a triangle matrix of pairwise linkage disequilibrium. The intensity of red shading indicates the level of linkage disequilibrium (*r*^2^) between variants.

To further confirm the association between natural variations of *TaPRR1* and yield-related traits, we performed an association analysis by retrieving genotype data spanning 1-Mb regions upstream and downstream of *TaPRR1* using the “Axiom Wheat 660K Genotyping Array” scanning 681 natural population (Supplementary Table S7). For *TaPRR1-A1*, there were 16 SNP markers spanning the physical position from 428,384,625 to 430,343,495 bp. Overall, *TaPRR1-A1* and 11 other SNPs exhibited significant association with HD and SL (*P* < 0.05) (Supplementary Table S9). *TaPRR1-A1* was in strong linkage disequilibrium (*r*^2^ > 0.5) with other significant variants, creating a linkage disequilibrium block flanking *TaPRR1-A1* (Figure 6A). Similar to *TaPRR1-A1*, *TaPRR1-B1* showed significant association with PH and TKW (*P* < 0.0001) (Supplementary Table S9) and was in high linkage disequilibrium with other significant SNPs (*r*^2^ > 0.5) (Figure 6B). For *TaPRR1-D1*, there were 35 SNP markers (Supplementary Table S9) and *TaPRR1-D1* was significantly associated with PH and TKW (*P* < 0.0001) (Figure 6C). It was also noticeable that the region of *TaPRR1-D1* coincided with several significantly associated SNPs (Figure 6C). Thus, *TaPRR1* possibly confers pleiotropic effects on agronomic traits.

### Geographical Distribution Characteristics of *TaPRR1* Haplotypes

In order to determine the distribution characteristics of *TaPRR1*, we investigated the distribution frequency of different *TaPRR1* haplotypes using 177 accessions from five continents including 15 countries.

**FIGURE 7:**
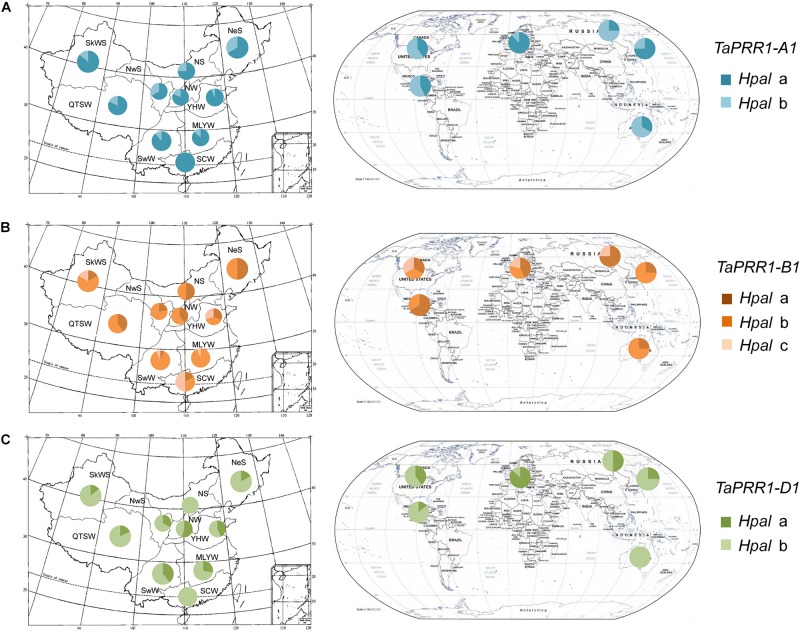
Geographic distributions of *TaPRR1* haplotypes. **(A)** Geographic distributions of *TaPRR1-A1*. **(B)** Geographic distributions of *TaPRR1-B1*. **(C)** Geographic distributions of *TaPRR1-D1*. The Chinese wheat planting areas were divided into ten major agro-ecological production zones (SkWS, Sinkiang Winter-Spring Wheat Region; QTSW, Qinghai-Tibet Spring-Winter Wheat Region; NeS, Northeastern Spring Wheat Region; NwS, Northwestern Spring Wheat Region; NS, Northern Spring Wheat Region; NW, Northern Winter Wheat Region; YHW, Yellow and Huai River Winter Wheat Region; MLYW, Middle and Low Yangtze Valley Winter Wheat Region; SwW, Southwestern Winter Wheat Region; and SCW, South China Winter Wheat Region). Other areas included Asia, Europe, North America, South America, and Oceania. Pie charts represent the proportional distribution of different haplotypes.

The Chinese wheat planting areas were divided into ten major agro-ecological production zones according to climatic characteristics, topography and soil type, planting system, and cultivation characteristics (Jin, 1983). We investigated the distribution frequencies of different *TaPRR1* haplotypes and found different distribution characteristics (Figure 7). For *TaPRR1*-*A1*, *Hapl*a accounted for a larger proportion than *Hapl*b in all ten ecological areas, which suggests *Hapl*a varieties are photoperiod insensitive and can adapt to a wide range of geographical conditions (Figure 7A). For *TaPRR1*-*B1*, *Hapl*b accounted for the highest proportion, followed by *Hapl*a and *Hapl*c in all ten ecological areas except SCW (South China Winter Wheat Region, where *Hapl*c accounted for the largest ratio) (Figure 7B). The cause of the above phenomenon may be that SCW is located in a low-latitude area with comparatively shorter day lengths as well as high temperatures in summer than other areas, and *Hapl*c is more suitable and adaptable for this climate compared to other haplotypes. For *TaPRR1*-*D1*, the proportion of *Hapl*b was higher than that of *Hapl*a (Figure 7C). It is worth noting that the haplotypes with the highest proportions in the B and D subgenomes do not carry favorable alleles. Therefore, the discovery of favorable alleles provides valuable theoretical reference for molecular breeding of wheat in these regions.

The distribution characteristics of different *TaPRR1* haplotypes in other countries were different from those in China (Figure 7). For *TaPRR1-A1*, *Hapl*a accounted for a larger proportion than *Hapl*b in Asia and Europe, whereas other regions were dominated by *Hapl*b. For *TaPRR1-B1*, *Hapl*b accounted for the highest proportion in Asia and Oceania. For *TaPRR1-D1*, it is noteworthy that *Hapl*a was predominant in Europe, which was opposite to situations in other regions.

### Differentiation of Genetic Variations of *TaPRR1* Between Landraces and Modern Cultivars

**FIGURE 8:**
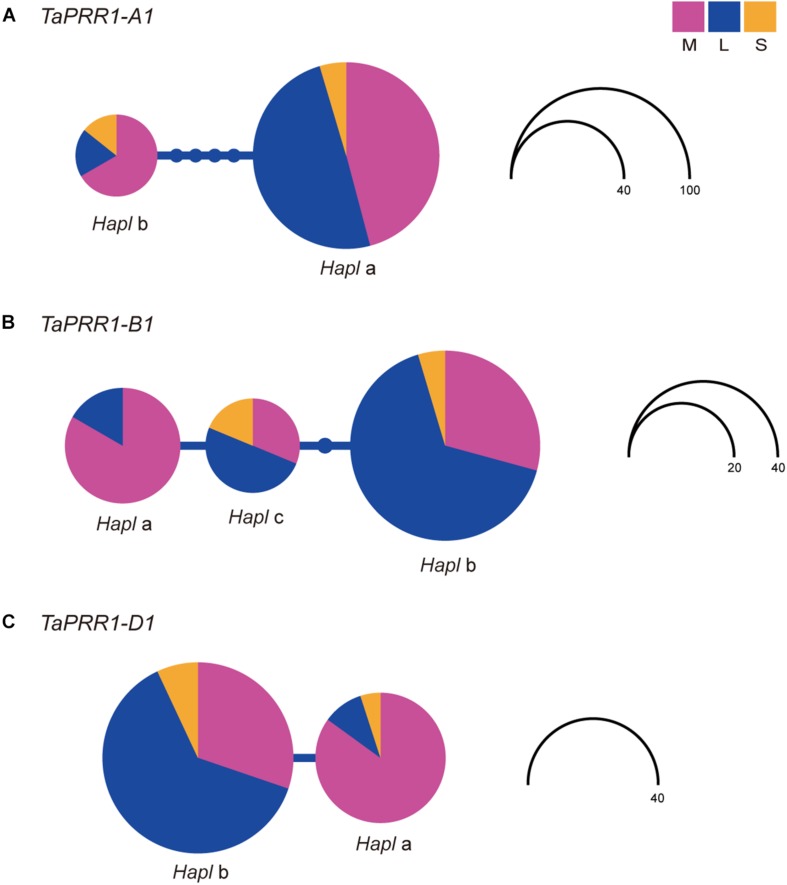
Haplotype network of *TaPRR1-A1*, *-B1*, and *-D1*. Coding regions of *TaPRR1-A1*, *-B1*, and *-D1* plus 2kb upstream of the coding region of *TaPRR1-D1* were used for haplotype analysis. A haplotype network was drawn using haplotype viewer (http://www.cibiv.at/~greg/haploviewer). One circle represents a haplotype. The node above the line indicates the number of base substitutions needed to get from one haplotype to another. Circle size represents the number of haplotypes. Different colors represent different subgroups. Purple, blue, and orange pie charts represent modern cultivars, landraces, and synthetic hexaploid wheat, respectively. Haplotype networks of *TaPRR1-A1*, *-B1*, and *-D1* are shown in **(A)**, **(B)**, and **(C)**, respectively.

To determine the differentiation degree of genetic variation of *TaPRR1*, we analyzed the distribution frequencies of haplotypes of *TaPRR1-A1*, *-B1*, and *-D1* in landraces, modern cultivars, and synthetic hexaploid wheat lines (Supplementary Table S4). Haplotype network analysis showed that genetic variations in *TaPRR1* were different between landraces and modern cultivars (Figure 8). However, no differentiation existed between synthetic hexaploid wheat and landraces/modern cultivars. For *TaPRR1-A1*, *Hapl*a was predominant in landraces. However, the percentage of *Hapl*b increased from 5.1% in landraces to 23.3% in modern cultivars (Figure 8A). For *TaPRR1-B1*, the percentage of *Hapl*a, which represents a favorable haplotype able to reduce PH and increase TGW, was increased from 7.3% in landraces to 42.9% in modern cultivars (Figure 8B). Similarly, the percentage of *TaPRR1-D1-Hapl*a was greatly increased in modern cultivars (5.1% in landraces versus 58.6% in modern cultivars) (Figure 8C). AMOVA revealed significant differences in haplotype distribution frequency between landraces and modern cultivars in *TaPRR1-A1* (*F*_*st*_ = 0.07; *P* < 0.05), *TaPRR1-B1* (*F*_*st*_ = 0.21; *P* < 0.05), and *TaPRR1-D1* (*F*_*st*_ = 0.43; *P* < 0.05), respectively (Supplementary Table S10). According to the *F*_*st*_ value, we speculated that *TaPRR1-A1* showed a moderate degree of genetic differentiation whereas *TaPRR1-B1* and *-D1* showed a high degree of genetic differentiation between landraces and modern cultivars.

## Discussion

Wheat is a widely cultivated crop that has formed diversified ecological types adapted to different conditions worldwide (Worland and Snape, 2001). With the continuous enrichment of wheat resources as well as extensive exchange of new cultivars across the world, the ecological types of wheat are becoming more diversified. Developmental characteristics affect the regional distribution, introduction range, and utilization of wheat, and form an important basis for research into wheat cultivation and adaptation. Wheat is a long-day crop and its flowering is accelerated by a long photoperiod. Under short days, heading is delayed in photoperiod-sensitive cultivars (Klaimi and Qualset, 1973). Mutation of common hexaploid wheat into photoperiod-insensitive accessions enables it to adapt to different environmental changes (Worland et al., 1998).

### Homeolog-Specific Functions of *TaPRR1*

The circadian clock regulates diverse aspects of plant growth and development; it not only confers daily rhythms in growth and metabolism, but also interacts with signaling pathways involved in plant responses to the environment (Harmer, 2009). *PRR1* is a key component of the plant circadian clock, and the maintenance of its rhythmic expression is essential for proper functioning of the circadian clock (Somers et al., 1998; Más, 2008). In the present study, we analyzed polymorphisms in *TaPRR1* among diverse wheat germplasm. Based on the observed polymorphisms, we identified two, three, and two haplotypes for *TaPRR1-A1*, *TaPRR1-B1*, and *TaPRR1-D1*, respectively. Association analyses supported an association between haplotypes of *TaPRR1* and agronomic traits under multiple environmental conditions. However, it is noteworthy that the functional strength of *TaPRR1-A1*, *TaPRR1-B1*, and *TaPRR1-D1* was different. Specifically, *TaPRR1-A1* was related to the photoperiod sensitivity of wheat; accessions with *Hapl*a had an earlier HD and showed a photoperiod-insensitive phenotype. *TaPRR1-A1* was also weakly associated with SL. Different from the characteristics controlled by *TaPRR1-A1*, *TaPRR1-B1*, and *-D1* had similar functions and were significantly associated with PH and TGW. However, *TaPRR1-D1* showed a stronger function than *TaPRR1-B1*. Interestingly, different from *TaPRR1-A1*, neither *TaPRR1-B1* nor *-D1* was associated with heading time. Therefore, although *TaPRR1* belongs to the circadian clock genes, its function is not limited to the regulation of heading stage in wheat, but also affects other yield-related traits. In addition, *TaPRR1* is reported to regulate flag leaf angle, which is significantly correlated with the photosynthetic ability of a plant (Zhao et al., 2016). Moreover, it is worth noting that there are several previously reported yield-related quantitative trait loci (QTLs) (Wang et al., 2012; Edae et al., 2014; Zou et al., 2017) in the region of *TaPRR1*. Therefore, *TaPRR1* might co-locate with these QTLs and confer complex pleiotropic effects on agronomic traits.

### Significance of the *PRR* Gene Family in Whea**t**

Although wheat *PRR* family members vary in length, they all contain two conserved domains, a REC domain at the N-terminus and a CCT motif at the C-terminus. PRRs have conserved protein structure, suggesting that they might have similar biological functions. *PRR* gene family members in *Arabidopsis* function as central oscillators of the circadian clock. PRR3 is a vascular regulator of PRR1 stability, which perturbs PRR1 interaction with ZTL (ZEITLUPE) that targets PRR1 for proteasome-dependent degradation (Para et al., 2007). PRR9, PRR7, and PRR5 act as transcriptional repressors of CCA1 and LHY, constituting the molecular mechanism accounting for the role of these proteins in the feedback loop of the circadian clock (Nakamichi et al., 2010). In wheat, *PRR* family members, including “Green Revolution” gene *Ppd-1* (*PRR37*) and its homologous gene *TaPRR73* have been studied extensively (Beales et al., 2007; Wilhelm et al., 2009; Guo et al., 2010; Seki et al., 2011; Díaz et al., 2012; Nishida et al., 2013; Sun et al., 2014; Zhang et al., 2016; Arjona et al., 2018). However, research on *TaPRR1*, the core member of the circadian clock, has lagged behind. Both this study and previous studies have shown that *TaPRR1* affects heading time of wheat. In our study, we also found that *TaPRR1* affects agronomic traits including PH, TGW, and SL. It is worth noting that in the majority of agro-ecological production zones of China, varieties with favorable alleles of *TaPRR1* did not prevail, although the favorable haplotypes were positively selected during the wheat breeding process. Undoubtedly, the discovery of favorable alleles of *TaPRR1* and the development of molecular markers are of great significance for marker-assisted breeding of wheat. In addition, a pair of homologous genes, *TaPRR*59 and *TaPRR*95, is also worth studying. Phylogenetic analysis showed that *TaPRR1* has a close relationship with *TaPRR95* and *TaPRR59*, and spatiotemporal expression analysis showed that these genes also have similar expression patterns. Considering the important effects of *TaPRR1* on agronomic traits, *TaPRR95* and *TaPRR59* may be worth further investigation in the future.

### Epigenetic Modification Characteristics of *TaPRR1*

Previous studies have shown that *TaPRR1* is regulated by tae-miR408, a form of epigenetic regulation (Zhao et al., 2016). Interestingly, *Ppd-B1* was also reported to be regulated by epigenetic modification (DNA methylation), and DNA methylation affects the transcriptional levels of this gene (Sun et al., 2014). To further determine whether *TaPRR1* is regulated by other epigenetic factors, we retrieved DNA methylation and histone modification data for the *TaPRR1* gene (Appels et al., 2018) from The Triticeae Multi-omics Center website. DNA methylation represents an important epigenetic mechanism of gene regulation and is generally associated with the repression of gene transcription (Chan et al., 2005; Reddington et al., 2013). Histone methylation is an important post-translational modification. In general, H3K4me3 modification is usually enriched in the activated promoter region and relates to the activation of gene expression whereas H3K27me3 is associated with transcriptional inhibition (Kouzarides, 2007; Vaillant and Paszkowski, 2007). According to ChIP-seq data, the DNA methylation level of the gene body region of *TaPRR1* was lower than that of the promoter region (Supplementary Figure S2). Interestingly, *TaPRR1-A1*, *TaPRR1-B1*, and *TaPRR1-D1* had distinct DNA methylation patterns. The methylation level of *TaPRR1-A1* was the highest whereas that of *TaPRR1-B1* was the lowest. In terms of histone modification, the degree of *TaPRR1* histone modification on different subgenomes was roughly the same (Supplementary Figure S2). H3K4me3 was mainly concentrated near the transcriptional start site, whereas H3K27me3 was enriched over the entire genic region. Therefore, epigenetic modification, especially DNA methylation, might play an important role in the regulation of *TaPRR1*, which is also a key research object for the future.

## Data Availability Statement

All datasets generated for this study are included in the article/Supplementary Material.

## Author Contributions

HS, ZG, and JJ designed the research. HS, WZ, YW, FC, and CZ performed the experiments. HS, YW, and LG analyzed the data. HS and ZG wrote the manuscript. JJ revised the manuscript. All authors read and approved the manuscript.

## Conflict of Interest

The authors declare that the research was conducted in the absence of any commercial or financial relationships that could be construed as a potential conflict of interest.

## Footnotes

1 https://www.uniprot.org/

2 http://wheat.pw.usda.gov/ggpages/topics/Wheat660_SNP_array_developed_by_CAAS.pdf

3 http://cmpg.unibe.ch

4 http://202.194.139.32/

5 http://www.wheat-expression.com/

6 https://www.dna.affrc.go.jp/PLACE/
