# Supplementary material for: The Circadian Clock Gene, TaPRR1, Is Associated With Yield-Related Traits in Wheat (Triticum aestivum L.)
Source: Front Plant Sci. 2020 Mar 12;11:285. doi: 10.3389/fpls.2020.00285 (PMC7080851; doi:10.3389/fpls.2020.00285)
Supplement: Supplementary file 1 [file Image_1.PDF]

Figure S1

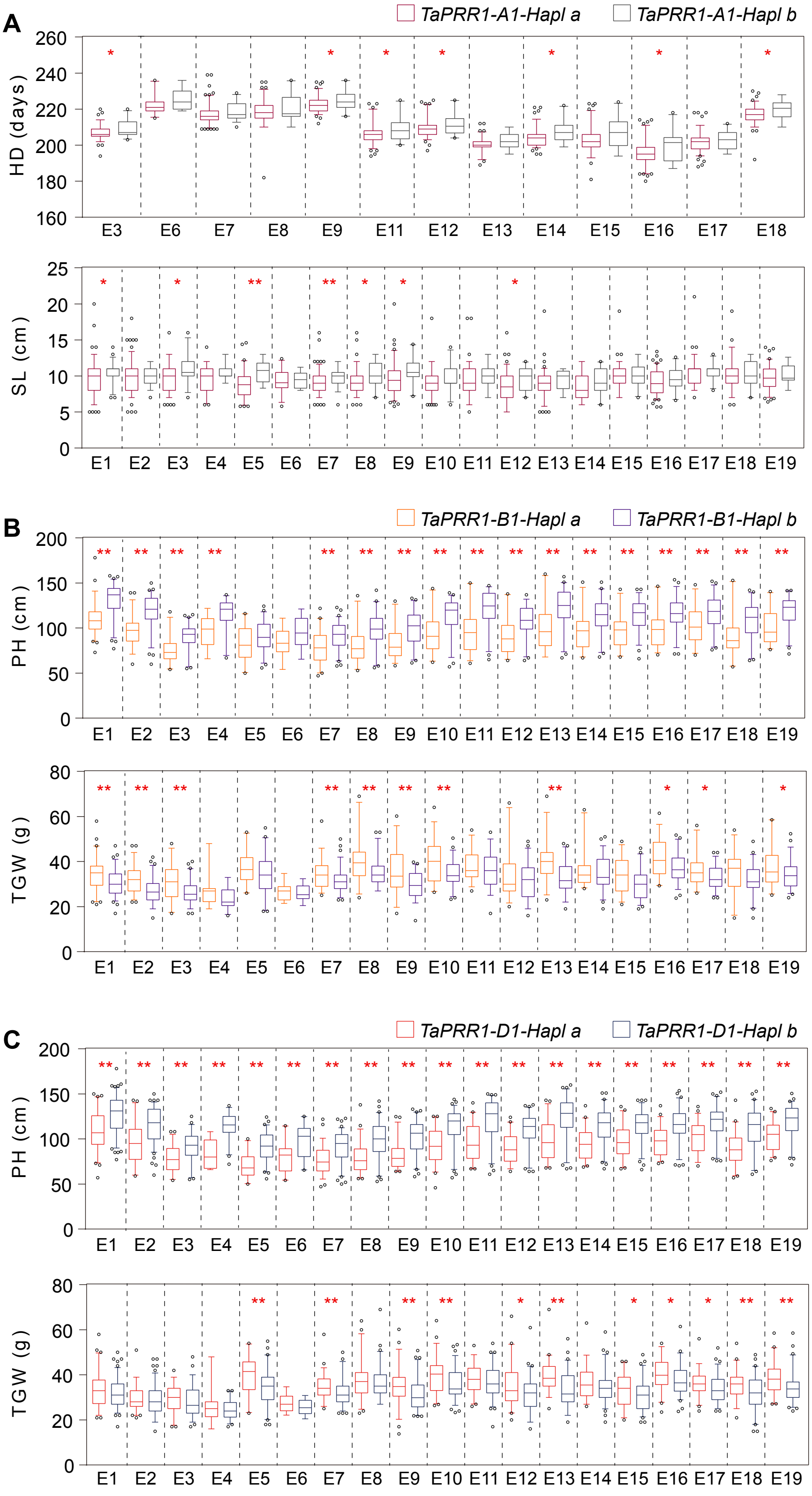

Figure S1 Phenotypic comparisons of large-effect haplotypes of *TaPRR1* homeologs in nineteen environments. **(A)** Comparison of heading date and spike length of different haplotypes of *TaPRR1-A1*.**(B)** Comparison of plant height and thousand grain weight of different haplotypes of *TaPRR1-B1*. **(C)** Comparison of plant height and thousand grain weight of different haplotypes of *TaPRR1-D1*. Data for different phenotypic traits are represented by boxplots. The horizontal solid line represents the median. The upper and lower edges of the box, respectively, represent the upper and lower quartile.The short thin lines represent the maximum and minimum values. The circle represents an outlier. **P* < 0.05, ***P* < 0.01.
