# Supplementary material for: The Circadian Clock Gene, TaPRR1, Is Associated With Yield-Related Traits in Wheat (Triticum aestivum L.)
Source: Front Plant Sci. 2020 Mar 12;11:285. doi: 10.3389/fpls.2020.00285 (PMC7080851; doi:10.3389/fpls.2020.00285)
Supplement: Supplementary file 2 [file Image_2.PDF]

Figure S2

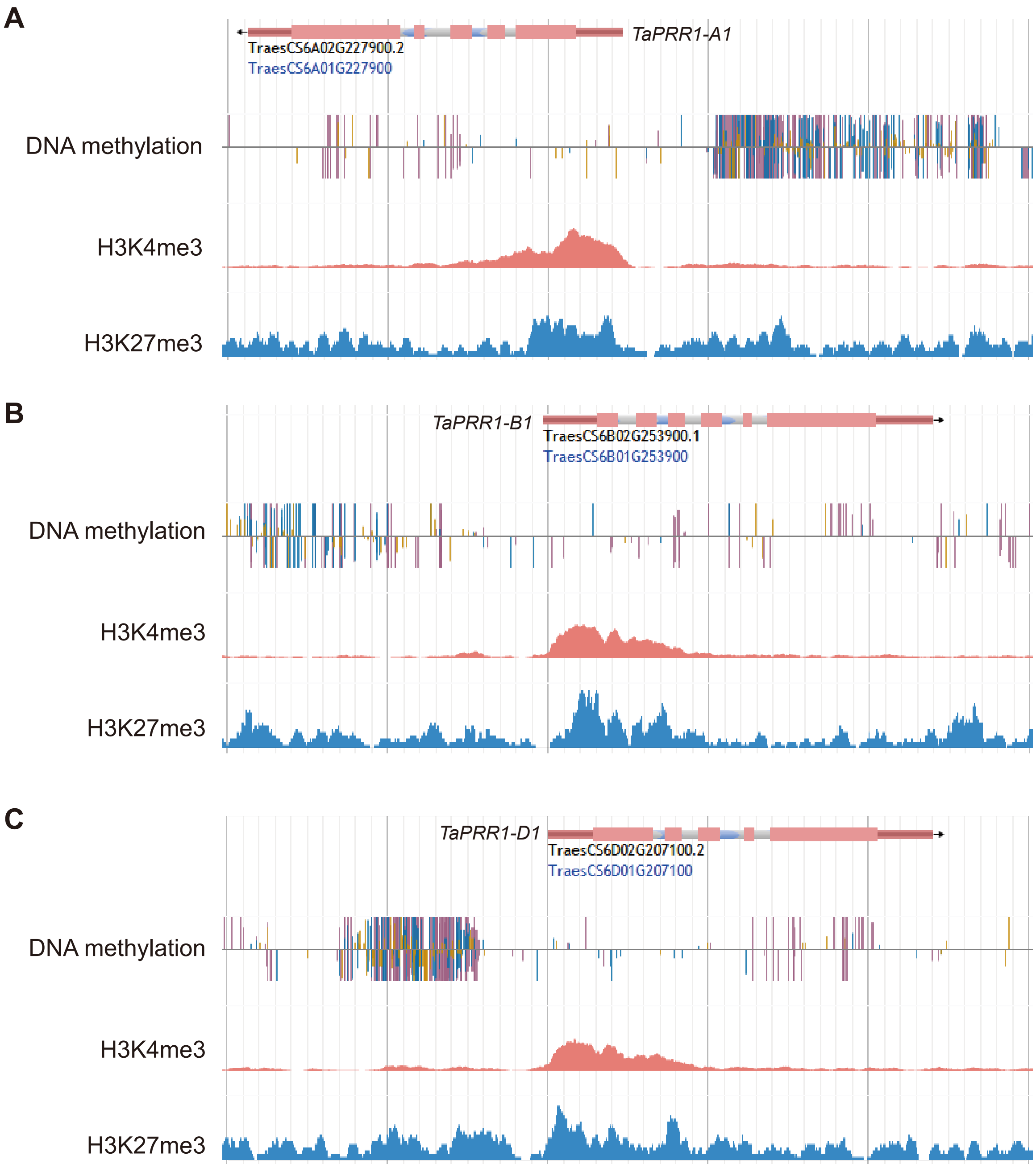

Figure S2 Epigenetic modification characteristics of *TaPRR1*. DNA methylation **(A)**, and H3K4me3 **(B)** and H3K27me3 **(C)** histone modifications of *TaPRR1* based on published data (http://202.194.139.32/).
